# Structural Correlation Coefficient for Polymer Structural Composites—Reinforcement with Hemp and Glass Fibre

**DOI:** 10.3390/polym17243295

**Published:** 2025-12-12

**Authors:** Mieczyslaw Scheibe, Magdalena Urbaniak, Andrzej Bledzki

**Affiliations:** 1 Faculty of Marine Engineering, Maritime University of Szczecin, 70-500 Szczecin, Poland; mscheibe@vp.pl; 2 Faculty of Mechanical Engineering and Mechatronics, West Pomeranian University of Technology in Szczecin, 70-310 Szczecin, Poland

**Keywords:** hemp fibre reinforced polymer composites, industrial hemp (*Cannabis sativa* L.), structural correlation coefficient, disposal, recycling

## Abstract

This article provides a multifaceted analysis of the feasibility, purposefulness, and legitimacy of the alternative use of industrial hemp (HF) fibres processed into fabrics and mats as multilayer reinforcement in polymer structural composites, potentially replacing glass fibres (GF) in various industries, including the production of recreational vessels (yachts and motorboats) and other floating products (buoys/floats/pontoons, etc.). Based on the results of physical, mechanical, and morphological tests of new polymer structural composites HFRP vs. GFRP and a comparative analysis of their properties, a structural correlation coefficient for HFRP was determined with respect to GFRP [W_K_ = 1.66 (6), provided that the grammage of reinforcement of the skin/shell of the selected floating object/structure is comparable]. This article presents the possibility of meeting stringent environmental protection requirements for the future safe recycling and/or disposal of products and their post-production waste manufactured from HFRP at the end of their service life. Fire tests of these new materials have shown that it is possible to use them completely (almost 100%) in the near future, mainly through energy recovery.

## 1. Introduction

The requirements of sustainable economic development necessitate the replacement of materials previously used in the manufacturing of technical products not only with lighter and more durable materials but also with materials that meet increasingly stringent environmental protection requirements. Adopted in Strasbourg on 16 January 2018, the pan-European strategy on plastics supports action in the field of environmental protection. The strategy aims to strengthen the position of the European industry in the context of increasing innovation in the design, manufacture, use, and recycling of products in the European Union. Compliance with current environmental requirements for the safe recycling and/or disposal of post-production waste from composite products and end-of-life (EOL) composite structures containing glass fibres (GF) is largely associated with the elimination of composite reinforcement in the form of *E*-glass fabrics and mats, which have been used for many years. Over the past few decades, technological developments in polymer materials, and, thus, polymer structural composites, have spread to almost all sectors of the economy, from the chemical industry in the area of everyday items, through the automotive industry, machinery, construction materials, electronics, aviation, armaments, shipbuilding, and even the space industry. Once technical products reach EOL status (e.g., wind turbine blades, car bodies, and rail vehicle bodies and interiors), the materials from which they are made are expected to be highly suitable for disposal by energy recovery. The implementation of the EU framework programme known as H2020 between 2013 and 2020 and the use of a universal technological process design based on technology readiness levels (TRLs) clearly indicate the need to resolve the global problem of disposal and/or recycling of end-of-life products and waste containing GF as quickly as possible. Furthermore, measures have been taken to initiate the manufacture of environmentally friendly products and to recycle existing products in order to protect the natural environment. The adoption and approval in December 2015 by the European Commission of a package on the circular economy, promoting innovative solutions to ensure an adequate level of environmental protection, also highlighted the need to address the global problem of disposal and/or recycling of mainly GF-reinforced recreational fibre-reinforced polymer (FRP) vessels being taken out of service. Yachts and motorboats, which are considered ‘luxury goods’, have been manufactured for about 50 years mainly from glass fibre-reinforced polymer (GFRP) reinforced with GF (approx. 98%), and less frequently from carbon fibre-reinforced polymer (CFRP) reinforced with carbon fibres (CF) (approx. 2%) [1]. The introduction of NPF fabrics, alongside or in place of GF fabrics as an alternative component for reinforcing polymer structural composites, is in line with the programme of pro-environmental measures recommended and required by the European Union. At the turn of the 20th and 21st centuries, the results of research conducted in the field of mechanical properties and industrial use of NPF, mainly hemp fibres (HF) and less frequently flax fibres (FF), in polypropylene (PP) and epoxy (EP) composites began to appear in the global specialist literature [2,3,4,5]. In the first decade of the 21st century, the results of research into the properties of HF and HF-reinforced composites as an alternative to GF reinforcement in polymer composites began to be published [6,7,8,9]. In the second decade of the 21st century, many research and development centres on different continents (North America, Europe, South East Asia) conducted research on the physical and mechanical properties of NPF-reinforced composites and presented factors and conditions that improve its mechanical properties [10,11]. At that time, the results of research on the physical and mechanical properties of HF were also published in relation to the technology of cultivation and processing of industrial hemp [12,13,14,15]. Moreover, at the turn of the second and third decade of the 21st century, the results of research conducted in the field of the influence of HF length on the properties of PP and EP composites were reported [16,17,18,19].

## 2. GFRP Floating Units and Their Disposal in Terms of Environmental Risks

**Figure 1:**
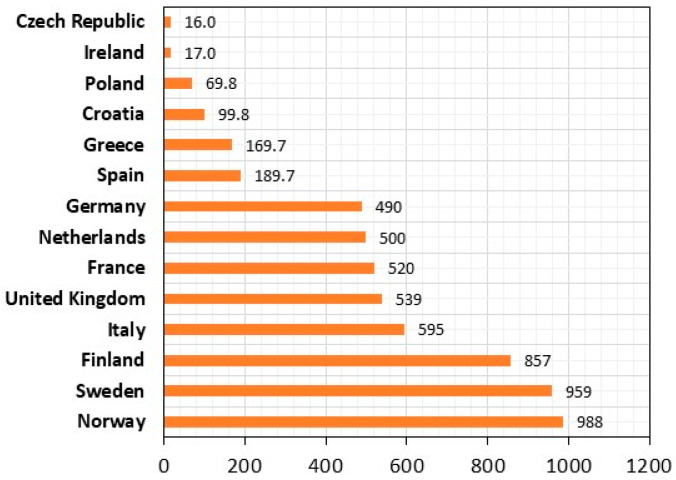
Number of recreational GFRP vessels in EU countries for the first quarter of 2025 (thousands) (own elaboration in conjunction with [20], 2025).

The annual growth in European production of composite GFRP vessels, observed for many years, has been estimated at approximately 6 million units in the European Union countries for the first quarter of 2025, of which approximately 988,000 units reside in Norway, approximately 959,000 units in Sweden units, and in Finland—approx. 857,000 units (Figure 1) [20].

According to the RC-1774 DMR (Dimension Market Research) Report, in 2023 the number of registered recreational vessels was 11.9 million in the USA; 2.6 million in Canada, and over 6 million in Europe. In 2022, over 180,000 units were registered in Japan, and 850,000 in Australia [21,22]. The service life of these vessels, as determined by the Nordic Council of Ministers—NORDEN, ranges from 35 to a maximum of 50 years, after which they should be disposed of. In reality, these vessels end up in landfills (USA), “anchorages” (Canada), or are incinerated at sea (Nordic countries). Global maritime organisations estimate that by the end of 2025, the amount of GFRP waste from end-of-life composite recreational craft worldwide will reach 2.58 million tonnes [21].

The issue of disposal and recycling of composite units is currently being addressed by global maritime organisations and associations: in Europe—mainly IMO, AQASS, and RYA, in the USA—NASBLA, in Canada—DML, and globally—NORDEN. In light of the applicable conventions and international regulations on environmental protection, meeting the current environmental requirements for the safe recycling and/or disposal of structures withdrawn from service after reaching EOL status is largely associated with the elimination of composite reinforcement, used for many years, in the form of glass fabrics and mats, mainly of the *E*-type. Due to the need to engage specialised technical resources and apply sophisticated chemical methods that adversely affect the natural environment, as well as the associated very high financial costs, these factors currently make complete disposal or effective recycling of products, their post-production waste, and other waste generated from GFRP material an exceptionally burdensome problem in the area of human environmental protection. Consideration has been given worldwide to all possibilities for the use of NPF-reinforced composites as an alternative to glass for reinforcing polymer structural composites. Materials of this type were used in various industries as early as the 1980s and 1990s. In the USA and Europe (mainly in Germany), NPFs such as cotton, flax, industrial hemp or jute are used in automotive components.

In the review of articles and scientific papers published worldwide at the turn of the 20th and 21st centuries on the possibilities of environmentally friendly uses of natural plant fibres (NPF), mainly bast fibres (jute, kenaf, industrial hemp, flax, and ramie), as an alternative to non-ecological synthetic glass fibres (GF) and reinforcement of polymer composites (FRP), no publications were found directly related to the possibility of their use in the boatbuilding and shipbuilding industries; in particular, FRP reinforced with bast fibres from plants found in Poland, i.e., industrial hemp and flax. Available published scientific studies usually present the results of research on the chemical properties (fibre content and others) and physical properties (density, water absorption and others) as well as the impact of the physical structure (porosity, thickness, length) of NPF bast fibres on the mechanical properties (tensile strength, flexural strength, and impact strength) of polymer composites reinforced with them. NPF research was conducted in the context of their potential application in polymer composites used in the chemical, textile, automotive, and building materials industries. The limited use of this type of reinforcement in other industries (e.g., shipbuilding, recreational craft construction (yachts and motorboats), and other floating products (buoys/floats, pontoons)) provides the basis for addressing the growing problem of disposal and/or recycling of these vessels and products, currently and in the future, when they are taken out of service (EOL). It should be noted that of the NPFs containing bast fibres, only industrial hemp and flax are naturally cultivated in Europe.

The replacement of difficult-to-dispose-of/recycling of end-of-life products and post-production residues of GF-reinforced FRP (type ‘*E*’ and ‘*S*’) with new and easily disposable NPF-reinforced FRP materials is dictated by the rapid global increase in landfills of unwanted and environmentally hazardous composite waste materials containing synthetic glass fibres. It should be noted that global production of GFRP products is gradually increasing year after year, with **the energy required to produce 1 tonne of GF amounting to 30.0 GJ/1 tonne and the price of 1 tonne of raw material ranging from 1600 to 3300 (average 2450) USD**. In the context of the high energy costs involved in GF production and the negative environmental impact of GFRP products reaching EOL status and their waste, administrative and legal measures to protect the natural environment were taken as early as the second decade of the 21st century, mainly in the EU and the USA. One of these measures is to reduce global production of GFRP products in favour of natural fibre polymer composites (NFPC). In view of the need to adopt restrictive EU legislation on this issue, measures have been taken in Poland to reduce the production of GFRP-based products in the future and to replace them with NFPC-based products. To this end, narrowing down the above issue to the areas of the boatbuilding and shipbuilding industries, technical universities in Szczecin/Poland have undertaken relevant basic research on the production of a new NPF-reinforced FRP.

An important criterion when considering the selection of fibre plants, in the context of their industrial applications and the technical use of their fibres, is their natural occurrence in a specific geographical region of the world. With regard to the European continent (Central Europe) and its climatic conditions, there are two fibre-producing plants: **flax and fibre hemp**. The basic criterion for selecting new NPF-reinforced FRPs for testing in shipbuilding applications was that they meet the following conditions:

(a) These crops are grown in Poland: **industrial hemp and flax**;
(b) Agricultural costs and growth in the area under cultivation of these plants in Poland: **industrial hemp: cheap, year-on-year growth in the area under cultivation and flax; expensive, stable area under cultivation at a certain level**;
(c) Impact on degraded soil: **industrial hemp—positive impact on degraded soil in the process of regenerating its structure and fertility by increasing organic matter content, improving microbial activity and regulating pH, minimal use of artificial fertilisers and no need for full-scale irrigation and flax; requires significant use of artificial fertilisers and full-scale irrigation**;
(d) Global raw material price in 2022 (USD/1 tonne): **industrial hemp fibres, 1000–1900 (average 1450), and flax fibres: 2100–4200 (average 3150)**;
(e) Energy required to produce 1 tonne of fibre (GJ/1 tonne): **industrial hemp fibre, 4.0, and flax fibre, 4.5**;
(f) Distinctive physical, chemical, and mechanical properties of plant fibres: **industrial hemp and flax—comparable**.

A general analysis of agricultural opportunities and conditions in Poland, including economic and financial aspects, as well as a detailed comparative analysis of the physical, chemical, and mechanical properties of industrial hemp and flax fibres, clearly pointed to hemp as the best reinforcement for FRP. The final selection of hemp led to comprehensive physical and mechanical testing, environmental testing (the reaction of composites to fire and their disposal by energy recycling), and morphological testing (SEM method), performed using the HLU method (manual method) in the production process of a series of polymer structural composites containing a varying number of reinforcement layers. It should be noted that the primary objective of the global use of FRPs reinforced with NPF bast fibres and, in particular, in Poland FRPs reinforced with industrial hemp bast fibres, is the possibility of their comprehensive (nearly 100%) utilisation in the near future, mainly through energy recovery. Taking into account the economic considerations of growing these plants, in light of the analysis of these two plants in terms of agriculture, ecology, and economics, as well as a detailed analysis of the physical and mechanical properties of their fibres, their production volume (thousands of tonnes), and the price of raw material/mat/fabric (USD/1 tonne) and the energy required for their production (GJ/1 tonne), with regard to *E*-type glass fibres, the plant that fully and positively met all the conditions in the area of the aspects under consideration was selected from among them, i.e., the globally common **fibre hemp** (*Cannabis sativa* L.), also known as **industrial hemp** or **seed hemp** (**HF**) [23,24,25,26,27]. A comparative analysis of HFs in relation to GFs showed that HFs have lower density, comparable elongation and Young’s modulus, a lower raw material price, and **7.5 times less energy required to produce 1 tonne of HFs [GJ/1 t)** [15,23,27].

## 3. Research Work—Assumptions

This study aimed to perform the following:

1. Examination of the validity and advisability of replacing commonly used GFRP structural composites with a new HF fibre-reinforced composite material in the construction of selected composite vessels, in light of the applicable regulations concerning the need to counteract degradation and protect the environment;
2. Production of a new environmentally friendly material reinforced with natural plant fibres in the form of HF, intended for the construction of hulls for yachts, motorboats, and other selected composite vessels;
3. Completion of a comparative analysis of selected physical and mechanical properties of HF-reinforced polymer structural composites with varying amounts of reinforcement material, demonstrating their full recyclability using energy recovery methods;
4. Determination of the structural correlation coefficient **W_K_** for a new environmentally friendly construction material in relation to GFRP, subject to the condition of comparable weight of the hull plating of a composite vessel.

## 4. Research Methodology

**Figure 2:**
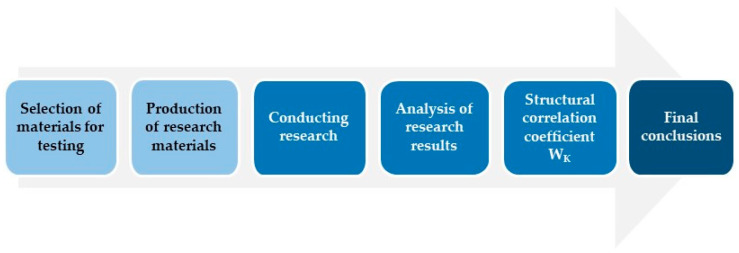
Block diagram of tasks and research carried out.

In order to carry out the designated tasks, the following algorithm was adopted (Figure 2):

The introduction of a new material containing industrial hemp fibres as a reinforcement for polymer structural composites into the shipbuilding industry is conditional on its meeting a number of different criteria, including the following: low density (specific weight), ability to absorb energy on impact, resistance to constantly changing environmental conditions, low price, and the ability to fully utilise composite post-production waste and technical products after they have reached their end-of-life status.

### 4.1. Selection of Materials for the Production of Composites for Testing

- DCPD polyester resin (known as improved polyester ‘yacht’ resin) with the trade name AropolTM M 604 TBR + Metox-50WR resin copolymerisation initiator + pro-adhesive agent in the form of maleic anhydride (MAH) in a ratio of 3 g per 100 g of resin;
- GF fabric—manufacturer: KROSGLASS/Krosno/Poland—weight 450 ± 27 g/m^2^;
- HF fabric—manufacturer: S.C. CAVVAS LIMITED S.R.L./Cluj-Napoca/Romania—weight 478 g/m^2^.

### 4.2. Production of Research Material

Control plates made of GFRP and new polymer structural composites reinforced with HF were manufactured using the hand lay-up (HLU) method in the production hall of TTS (Technologie Tworzyw Sztucznych Sp. z o.o. in Łozienica near Goleniów, Poland—manufacturer of yachts and motorboats). During the manufacturing process, the conditions specified in Points 4.2.2–4.2.4 were maintained [28]: constant room temperature within the range of 16–25 °C (average air temperature during the process 22 ± 1 °C); relative humidity below 70% (average air humidity during the process 66 ± 2%).

#### 4.2.1. Technology for the Production of Composite Materials Intended for Research

The composite materials intended for testing were made using the HLU method, which involves laying successive layers of reinforcement in an open mould at room temperature (here, seven base plates made of rigid fibreboard with a thickness of T = 6 mm; dimensions of W = 250 mm × L = 400 mm, constituting ½ the surface of the control board with dimensions of W = 400 mm × L = 500 mm × T mm, specified by PRS regulations, in accordance with Point 4.6.3 [28]) and then manually saturating them with resin with the addition of a resin copolymerisation initiator and a pro-adhesive agent in the form of maleic anhydride. The reinforcement layers were saturated with resin using rollers and brushes. After the resin was spread over the reinforcement, the composite was additionally rolled with a special roller to remove air bubbles.

#### 4.2.2. Composite Manufacturing Technique

In a room with a temperature of t = 22 ± 1 °C and humidity of 66 ± 2%, the base boards were placed in sequence on the laminating operating table (previously coated three times at 20 min intervals with a separating layer and seasoned at a temperature of t = 22 ± 1 °C for 72 h). For each of them, reinforcement material was arranged with an appropriate number of layers in the form of packages, x1 made of *E*-type glass fabric (6 sheets) and x6 made of industrial hemp fabric (in the following quantities: 3, 5, 7, 9, 11, 13 sheets), and the process of manufacturing composite control plates was carried out using the HLU method (Table 1).

#### 4.2.3. Production of Control Plates and Test Samples

Preparation of GF reinforcement formats (*E*-glass rowing fabric) and HF (industrial hemp fabric laid with the longer side parallel to the warp threads of the fabric) was performed by cutting the format materials (280 mm × 430 mm) from the fabric samples. After completing this process, they were left to cure for 72 h, and then all composite control plates were removed from the base plates and samples for testing were cut out using a water jet (*p* = 3950 bar). One GF-reinforced polymer composite was obtained as the base material GFRP, and six HF-reinforced polymer composites were obtained as a new polymer material named/designated **HFRP**.

### 4.3. Conducting Research

In order to determine the polymer properties of structural composites, base GFRP and new **HFRP** material, samples of these materials were subjected to the following:

- Physical testing (density, water absorption, reaction to fire, and environmental testing (disposal by energy recycling));
- Mechanical testing:
  1. Tensile strength testing of materials in accordance with EN ISO 527-2 [29] (using a universal testing machine SHIMADZU (Shimadzu, Tokyo, Japan) type AG-X plus (MWG − 20 kNA, CAP 20 kN, at the feed of 10 mm/min and at the temperature of 22 °C));
  2. Static bending strength testing of materials in accordance with EN ISO 178 [30] (using the universal testing machine as above, at the temperature of 22 °C);
  3. Charpy impact testing of materials in accordance with EN ISO 179-2 [31] on standard specimens without notches (using a pendulum hammer VEB Werkstoffpruefmaschinen Leipzig-Betrieb des VEB Fritz Heckert (Saxony, Germany) with a maximum impact energy of up to 50 J and up to 300 J).
- Morphological properties of materials (using a scanning electron microscope—SEM VEGA3 TESCAN, TESCAN, Brno, Czech Republic (beam voltage of 5 kV, scattered electrons detector)).

### 4.4. Analysis of Research Results

The analysis of the mechanical properties of the new **HFRP** polymer composite material in comparison to the base GFRP mainly concerns parameters that have a significant impact on the quality of this construction material, which can be used in the boatbuilding and shipbuilding sectors of the shipbuilding industry in the production of hulls (sides, decks, superstructures, and structural equipment) of selected vessels. Furthermore, in order to expand the analysis, empirically determined series of KE1 (10 reinforcement layers) and KE2 (12 reinforcement layers) composite samples were introduced (Table 1).

#### 4.4.1. Analysis of Tensile Strength Test Results for Composite Samples

**Figure 3:**
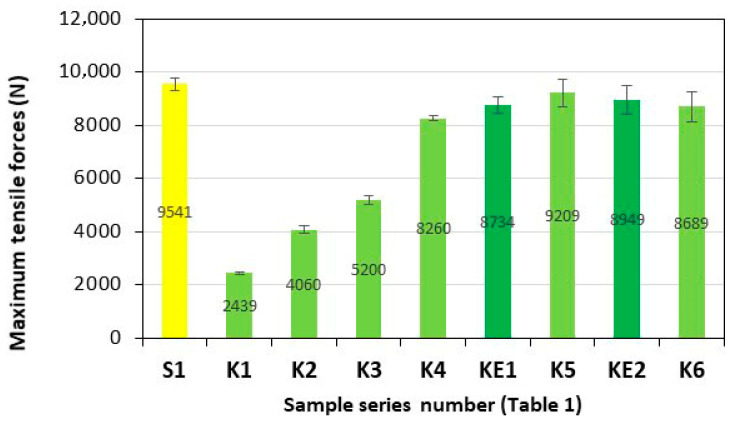
Maximum tensile forces of the tested composites. Yellow—with GF; light green—with HF; dark green—with HF empirically determined series of KE1 and KE2.

**Figure 4:**
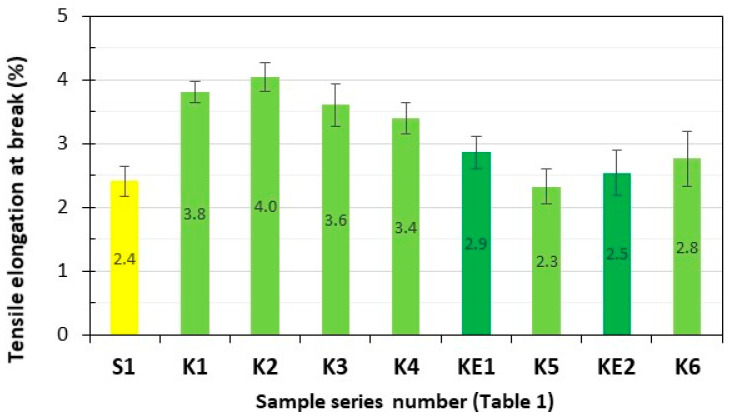
Tensile elongation at break of the tested composites. Yellow—with GF; light green—with HF; dark green—with HF empirically determined series of KE1 and KE2.

Series of composite samples, S1 (GFRP) and K1–K6 (**HFRP**), five pieces in each series, were subjected to tensile testing, and the results are presented in Figure 3 and Figure 4.

The maximum tensile force of the tested **HFRP** composites most similar to S1, i.e., series K5 (9209 N) and K6 (8689 N), are 3.5% and 8.9% lower, respectively, compared to the GFRP of the S1 series (9541 N).

Tensile elongation at break of the tested **HFRP** composites most similar to S1, i.e., series K5 (2.3%) and K6 (2.8%), are greater by 4.2% and 16.7%, respectively, in relation to the GFRP series S1 (2.4%).

#### 4.4.2. Analysis of the Results of Static Bending Strength Tests on Composite Samples

**Figure 5:**
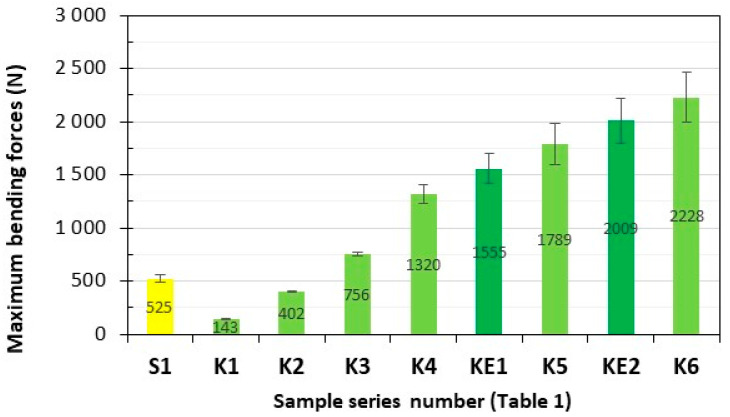
Maximum bending forces of the tested composites. Yellow—with GF; light green—with HF; dark green—with HF empirically determined series of KE1 and KE2.

**Figure 6:**
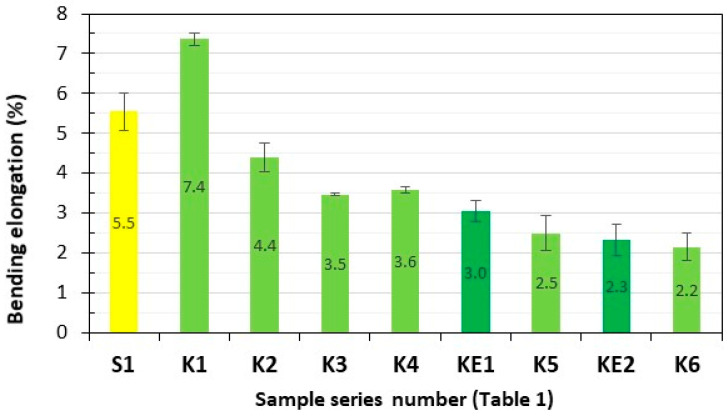
Maximum bending elongations of the tested composites. Yellow—with GF; light green—with HF; dark green—with HF empirically determined series of KE1 and KE2.

Series of composite samples, S1 (GFRP) and K1–K6 (**HFRP**), five pieces in each series, were subjected to static bending strength testing, and the results are presented in Figure 5 and Figure 6.

The maximum static bending forces of the tested **HFRP** composites most similar to S1, i.e., series K4 (1320 N) and K5 (1789 N), are 151% and 241% higher, respectively, compared to the GFRP of the S1 series (525 N).

The maximum bending elongations of the tested **HFRP** composites most similar to S1, i.e., series K4 (3.6%) and K5 (2.5%), are lower by 35% and 55%, respectively, in relation to the GFRP series S1 (5.5%).

#### 4.4.3. Analysis of Impact Test Results (Charpy Method) for Composite Samples

Charpy impact strength is a dynamic test that determines the ability of a material to withstand sudden impact loads and allows the brittleness threshold to be established and the material’s resistance to fracture under dynamic loading to be verified. The tests were performed in accordance with ISO 179-2 on standard samples without notches and each time consisted of measuring the energy absorbed during the impact test of a sample of a specific series of composites (S1 and K1–K6) in relation to a specific cross-section of the sample (cm^2^). The first series of impact tests on composite material samples S1 (GFRP) and K1–K6 (**HFRP**) was conducted after one month of storage in dry trays in environment **(0)** (air). In order to determine the impact of NaCl‰ content and other trace elements in specific environments, **(1)** (demineralised water), **(2)** (fresh water—Lake Miedwie/Poland/Żelewo—gc (geographical coordinates): 53.288862, 14.869285), **(3)** (brackish water—salinity 7.8‰—Baltic Sea/Poland/Dziwnow—gc: 54.029464, 14.762658), and **(4)** (salt water—salinity 38‰—Adriatic Sea/Croatia/Verudela Beach—gc: 44.833609, 13.832109), for the transfer of sudden dynamic loads by these materials, in accordance with ISO 15314 [32] (Methods of exposure to the sea: Method C involving exposure in which the specimens are completely immersed in water), all specimens were subjected to storage for a period of three months in representative aquatic environments **(1)–(4)**. Before immersing these samples, all sections and edges of the tested samples that were not protected against water seepage were properly secured with a DCPD matrix [23,33].

**Figure 7:**
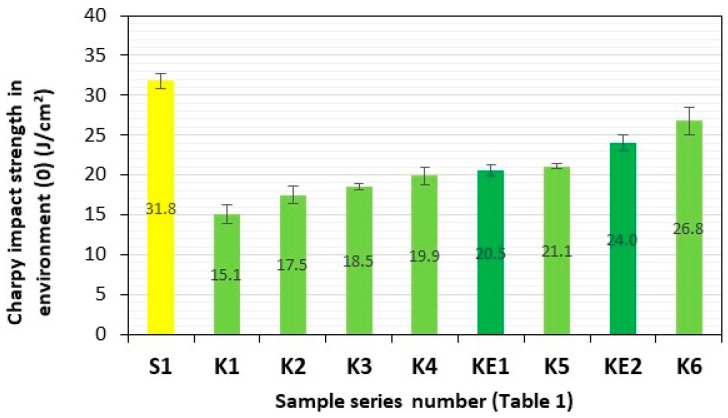
Charpy impact strength in environment **(0)** (air). Yellow—with GF; light green—with HF; dark green—with HF empirically determined series of KE1 and KE2.

**Figure 8:**
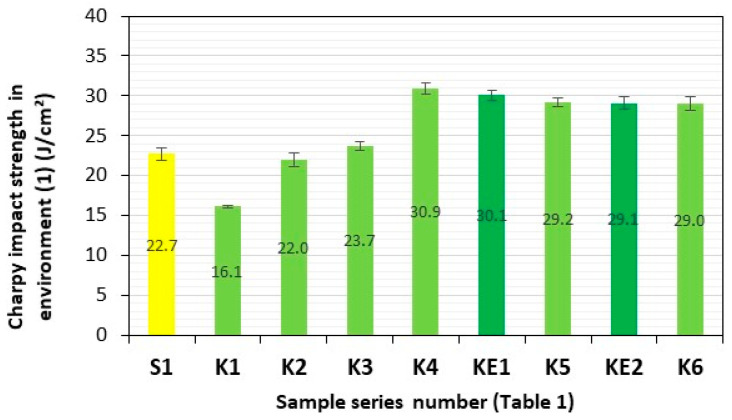
Charpy impact strength of composites in environment **(1)** (demineralised water). Yellow—with GF; light green—with HF; dark green—with HF empirically determined series of KE1 and KE2.

**Figure 9:**
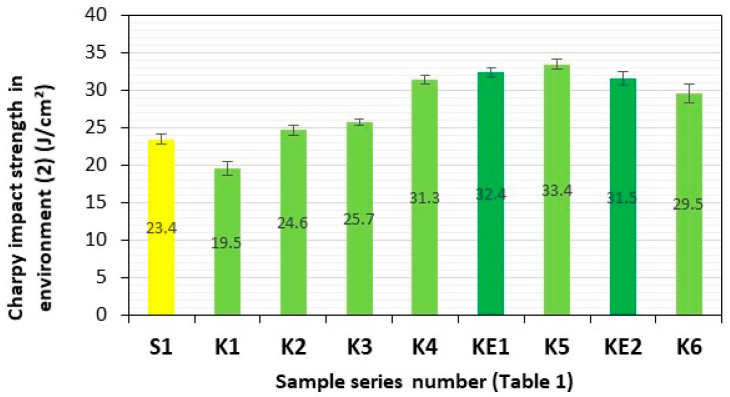
Charpy impact strength in environment **(2)** (fresh water—Lake Miedwie). Yellow—with GF; light green—with HF; dark green—with HF empirically determined series of KE1 and KE2.

**Figure 10:**
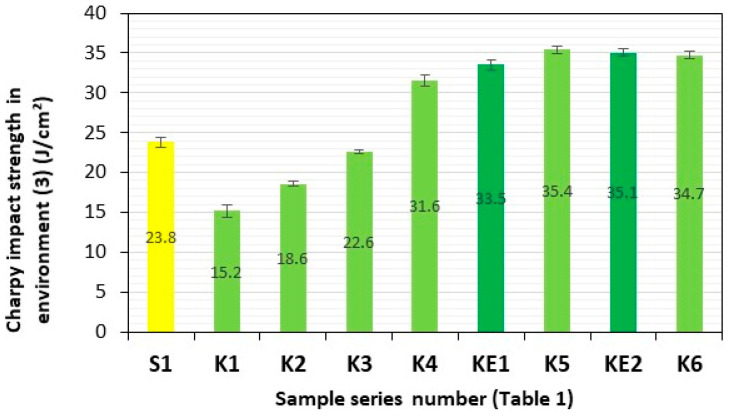
Charpy impact strength in environment **(3)** (brackish water—salinity 7.8‰—Baltic Sea). Yellow—with GF; light green—with HF; dark green—with HF empirically determined series of KE1 and KE2.

**Figure 11:**
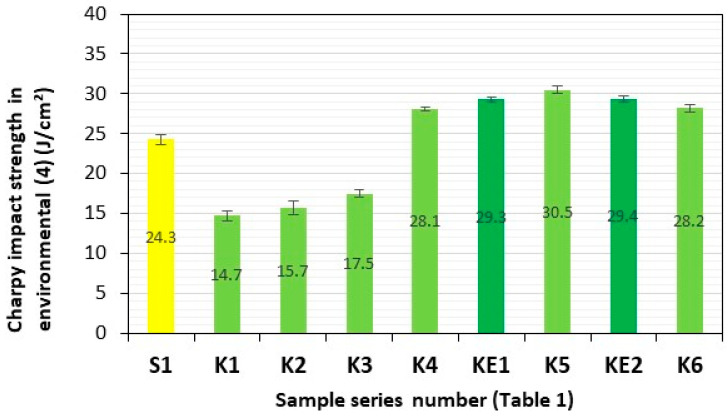
Charpy impact strength in environment **(4)** (salty water—salinity 38‰—Adriatic Sea). Yellow—with GF; light green—with HF; dark green—with HF empirically determined series of KE1 and KE2.

After a three-month period of storage of the samples of these composite materials in representative aquatic environments **(1)–(4)**, they were subjected to four consecutive series of impact tests. Series of composite samples, S1 (GFRP) and K1–K6 (**HFRP**), with five pieces in each series, were subjected to impact tests, and the results are presented in Table 2 and Figure 7, Figure 8, Figure 9, Figure 10 and Figure 11.

The impact strengths (Charpy method) of the tested **HFRP** composites in environment **(0)** (air) closest to S1, i.e., series K5 (21.1 J/cm^2^) and K6 (26.8 J/cm^2^), are lower by 34% and 16%, respectively, compared to the GFRP series S1 (31.8 J/cm^2^) (Figure 7).

The impact strengths (Charpy method) of the tested **HFRP** composites in environment **(1)** (demineralised water) most similar to S1, i.e., series K3 (27.0 J/cm^2^) and K4 (30.9 J/cm^2^), are higher by 19% and 36%, respectively, in relation to the GFRP series S1 (22.7 J/cm^2^) (Figure 8).

The impact strengths (Charpy method) of the tested **HFRP** composites in environment **(2)** (fresh water—Lake Miedwie) most similar to S1, i.e., series K3 (25.7 J/cm^2^) and K4 (31.3 J/cm^2^), are higher by 10% and 34%, respectively, in relation to the GFRP series S1 (23.4 J/cm^2^) (Figure 9).

The impact strengths (Charpy method) of the tested **HFRP** composites in environment **(3)** (brackish water—salinity 7,8‰—Baltic Sea) most similar to S1, i.e., series K4 (31.6 J/cm^2^) and K5 (35.4 J/cm^2^), are higher by 33% and 49%, respectively, in relation to the GFRP series S1 (23.8 J/cm^2^) (Figure 10).

The impact strengths (Charpy method) of the tested **HFRP** composites in environment **(4)** (salty water—salinity 38‰—Adriatic Sea) most similar to S1, i.e., series K4 (28.1 J/cm^2^) and K5 (30.5 J/cm^2^), are higher by 16% and 26%, respectively, in relation to the GFRP series S1 (24.3 J/cm^2^) (Figure 11).

#### 4.4.4. Analysis of the Suitability for Energy Recycling of a Selected Polymer Structural Composite

**Figure 12:**
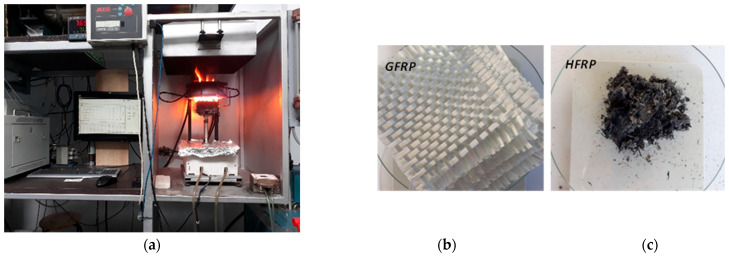
Energy recycling susceptibility of GFRP and **HFRP** polymer structural composites: (**a**) test rig for testing the reaction of materials to fire; (**b**) waste (after burning GFRP material) in the form of degraded E-type glass fabric; (**c**) waste (after burning **HFRP** material) in the form of ash.

The testing of the reaction of GFRP and **HFRP** samples to fire and their disposal by means of energy recycling was carried out using a cone calorimeter with an igniter in accordance with ISO 5660-1 [34], based on the International Code for the Application of Fire Test Procedures (FTP Code) adopted by the International Maritime Organisation (IMO) on 3 December 2010 by Resolution MSC.307(88). GF-reinforced GFRP samples and HF-reinforced **HFRP** samples were burned separately (Figure 12).

The method used to conduct these studies and the results obtained are described in detail in [23,33].

Analysis of the research results clearly showed the following:

1. The manufactured **HFRP** composite material subjected to energy recycling was almost completely utilised, i.e., **at a level of 98%**;
2. The waste produced after incineration of **HFRP** material consisted of **ash, amounting to 2% of its mass**;
3. The heat released during combustion of **HFRP** material was **183.0 MJ/m^2^**, while that of GFRP material was 48.8 MJ/m^2^ (test method—ISO 5660-1), which means that in the case of **HFRP** composite it **is 3.75 times greater** than the heat released during the combustion of the GFRP composite;
4. **HFRP** material can be used as energy fuel and the ash residue as an important component of effective natural fertiliser in line with EU preference for the CEco (*Circular Economy*).

### 4.5. Morphological Properties of HFRP Composites After Strength Tests

No descriptions of strength tests of **HFRP** structural polymer composites or the use of a scanning electron microscope (SEM) for testing them were found in the available technical literature. The morphological (structural) properties of polymer structural composites, the reference material GFRP and the new-generation material **HFRP**, were analysed on selected samples from the S1 series and the K1–K6 series after impact tests in mediums **(0)** and **(4)** and flexural strength tests in medium **(0)**. Observations of the structure of the external surfaces of the materials at their damage/destruction sites, resulting from mechanical testing of these samples, their internal properties (porosity, fibre alignment, and phase distributions), and detailed analyses of the matrix/reinforcement interface were conducted at variable magnifications in the range of 227x–1.42kx.

After strength testing of the S1 and K1–K6 series materials, SEM tests were performed on randomly selected samples in order to perform the following:

− Presentation of the general characteristics of the material structure with regard to the analysis of the matrix/reinforcement interface on a micron scale;
− Non-destructive visualisation of the internal features of the materials’ structure: porosity, fibre alignment, and matrix/reinforcement phase distributions;
− Detailed insight into the internal structure of the materials in areas of damage/destruction caused in the samples as a result of the mechanical test.

**Figure 13:**
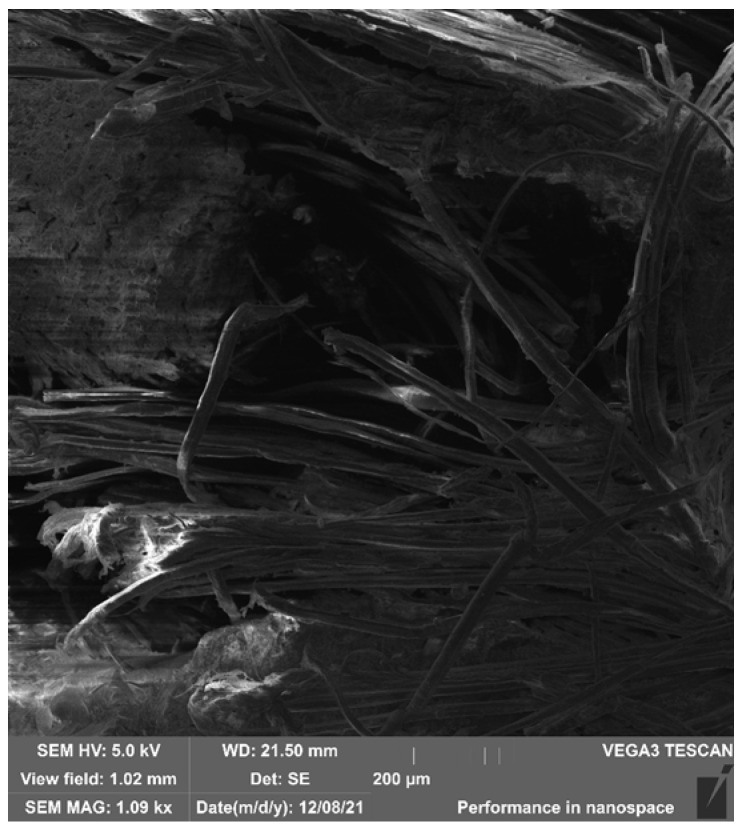
SEM image—magnification 1.09kx. General view of the fracture structure of the HFs in the **HFRP** composite (sample K6-Z2) after bending testing (Charpy method).

**Figure 14:**
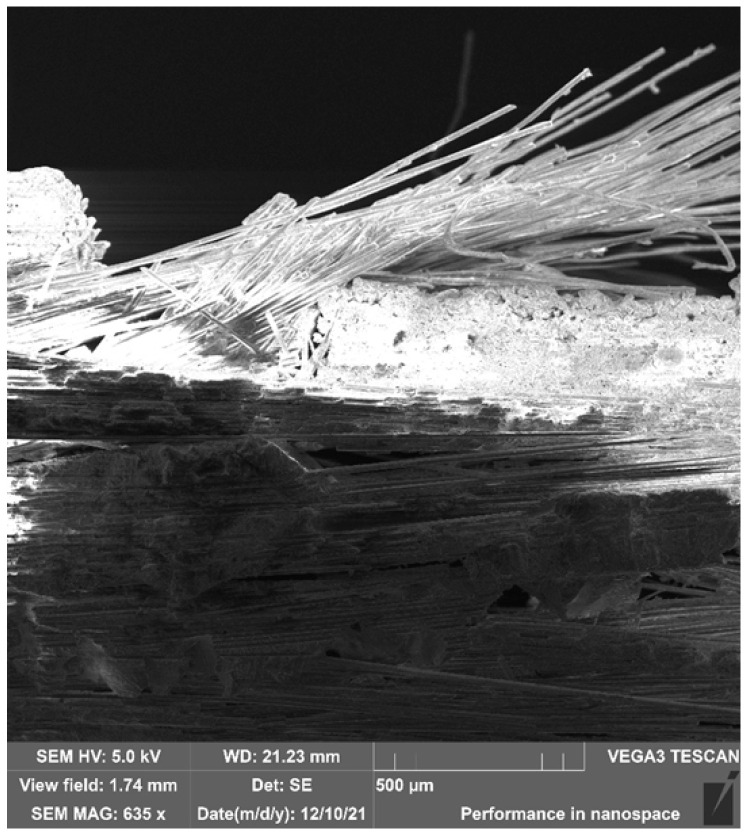
SEM image—magnification 635x. General view of the fracture structure of the GFs in the GFRP composite (sample S1-U1) after strength testing (Charpy method).

**Figure 15:**
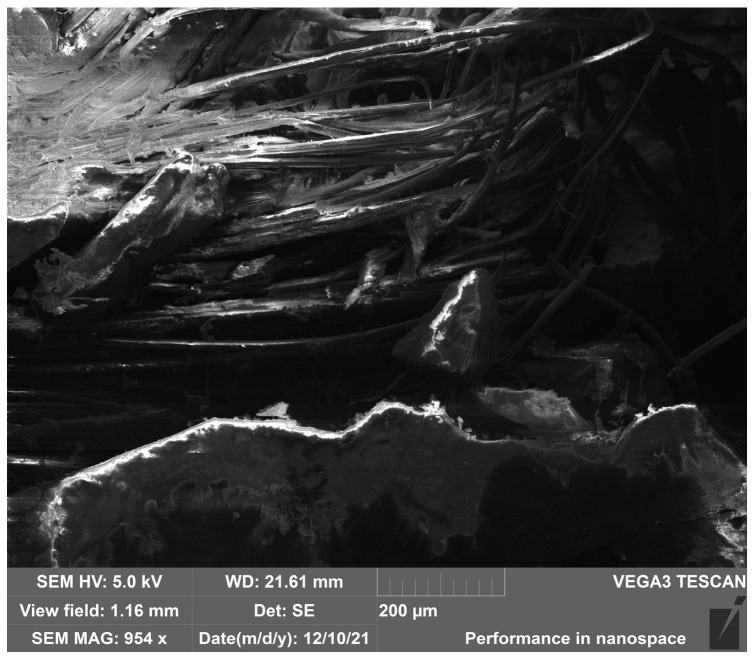
SEM image—magnification 954x. General view of the fracture structure of the HFs in the **HFRP** composite (sample K5-U5) after strength testing (Charpy method).

**Figure 16:**
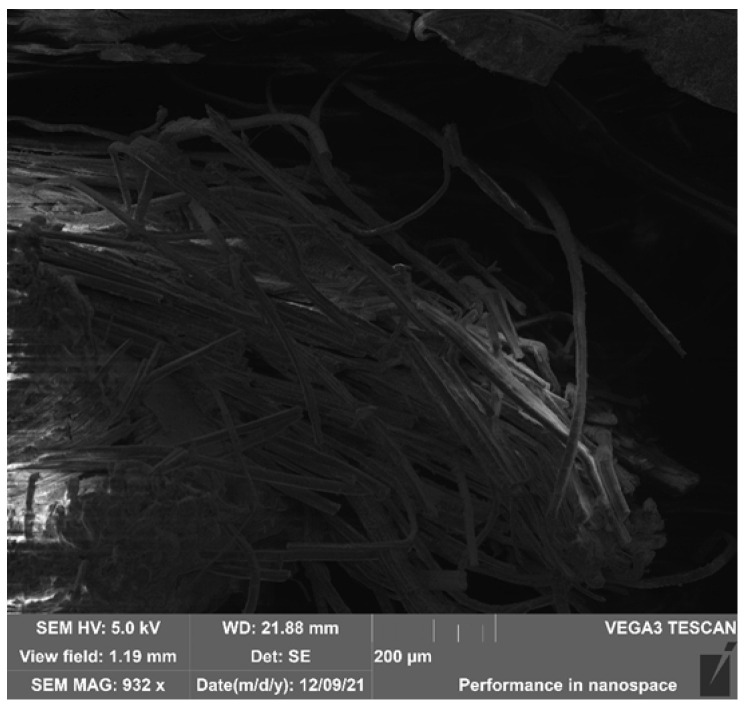
SEM image—magnification 932x. General view of the fracture structure of the HFs in the **HFRP** composite (sample K6-U1) after strength testing (Charpy method).

SEM imaging of **HFRP** composites of the K1-K6 and S1 series, after bending and impact testing (using the Charpy method), revealed few surface micropores and local inclusions in the form of numerous delaminations and chips and single fibres breaking the internal structure of the composites. Sample SEM micrographs of samples K6-Z2 (static bending test), S1-U1, K5-U5, and K6-U1 (Charpy impact test) of the **HFRP** composite are shown in Figure 13, Figure 14, Figure 15 and Figure 16.

SEM imaging of *E*-type glass fibres in sample S1-U1 after Charpy impact testing revealed significant inclusions in the form of numerous delaminations, chips, and individual fibres on the outer surface of the composite (Figure 14). The surface fractures of the material were brittle and sharp. No discontinuities in the matrix/reinforcement interface were observed, confirming the strong interlayer adhesion of the GFRP base composite components.

SEM imaging of selected samples from the HFRP composite series and their micrographic analysis revealed the presence of externally dishevelled surfaces and structurally internal microporous, partially deformed hemp reinforcement fibres. These fibre deformations occurred as a result of the fibre decortication process carried out at the stage of extracting them from this fibre plant for textile purposes. It should be clarified that the decortication process is carried out exclusively by mechanical means of fibre extraction through breaking, crushing, and beating the stems of this fibre plant.

The tests revealed a noticeable partial lack of coherence between the matrix and the hemp reinforcement fibres at the points where the warp and weft threads of the 100% hemp fabric (1/1 plain weave) intersect, which may affect the mechanical properties of these composites. During the tests, a structure with a multidirectional surface and intra-structural entanglement of porous hemp fibres was observed, as well as inclusions in the form of natural internal damage and interrupted warp structure occurring between individual fibres. Numerous inclusions in the form of empty spaces between fibres, air bubbles, and uneven phase distributions of the matrix/reinforcement interface were also observed.

### 4.6. Analysis of the Physical and Mechanical Properties of Tested Polymer Structural Composites

The results of the analysis of the physical and mechanical properties of the tested polymer structural composites, i.e., the new generation of **HFRP** composite meeting current environmental protection requirements, in relation to the commonly used, difficult-to-dispose-of GFRP composite are summarised in Table 3.

## 5. Structural Correlation Coefficient (W_K_) for Structural Polymer Composites HFRP vs. GFRP

Determination of the structural correlation coefficient (**W_K_**) (SCC **W_K_**) of the new **HFRP** composite material, in relation to the currently widely used base material GFRP, involves comparing the test results of all series of this material, determining their parameters that are most similar and that correspond to the parameters of the GFRP composite, and establishing the mutual proportion of the number of layers of their reinforcement.

As a result of **physical tests** [density (specific mass), water absorption, reaction to fire], **mechanical tests** [strength tests (tensile strength, flexural strength, impact strength in air and in various representative water media)], and **environmental testing** in the field of material disposal, preceded by verification of the mass and volume percentage of the reinforcement and **HFRP** matrix, a thorough analysis of all obtained test results was carried out.

From the many series of **HFRP** material considered in relation to the base GFRP material, the following series were selected, K4 (9 HF layers), K5 (11 HF layers), and K6 (13 HF layers), in the order of the best and most similar test results presented in the individual points of the research work (according to descriptive analysis using selection measures and standard deviations of the tested sample series [23]):
•Density of polymer structural compositeK4 and K5•Water absorption polymer structural compositeK4 and K5•Reaction to fire and suitability for disposal by means of energy recoveryK5•Tensile strength in the area of maximum tensile forces of compositesK5 and K6•Tensile strength in the area of compositeK5 and K6•Static bending strength in the area of maximum bending forces of compositesK5 and K4•Static bending strength in the area of composite elongationK5 and K4•Impact strength (Charpy method) of composite in environment **(0)** (air)K6 and K5•Impact strength (Charpy method) of the composite in environment **(1)** (demineralised water)K4 and K5•Impact strength (Charpy method) of the composite in environment **(2)** (fresh water (Lake Miedwie))K5 and K4•Impact strength (Charpy method) of the composite in environment **(3)** (brackish water (salinity 7.8‰—Baltic Sea))K5 and K4•Impact strength (Charpy method) of the composite in environment **(4)** (salt water (salinity 38‰—Adriatic Sea))K5 and K4

After a detailed analysis of the obtained test results, the empirical variant of the **KE1** series reinforced with **10** layers of 100% HF fabric was selected. The designated SCC **W_K_** for **HFRP** vs. GFRP, hence, the value of this coefficient adopted in the future for structural calculations, subject to the condition of comparable weight of the composite skin/shell of the selected floating object/structure, should be the following:

**W_K_ = 1.66 (6)**

It should be noted that the SCC **W_K_** correlation coefficient presented refers to a new generation of **HFRP** material containing reinforcement made from 100% industrial hemp fabric with a comparable reinforcement weight to traditional GFRP composite made from 100% *E*-glass fabric.

The fulfilment of the required thickness differentiation condition and the great freedom of choice of components included in the structure of the new construction material, in individual areas of the cladding of the structure being created, depending on its physical properties (hardness, density), strength properties (tensile strength, static bending strength, impact strength), and resistance to the environment affecting it, are additional advantages of their use in the manufacturing of large-size elements with ultimately defined complex product shapes.

The designated SCC **W_K_** should still be verified in application studies of alternative construction materials dedicated to selected technical products manufactured in the shipbuilding and boatbuilding industries.

## 6. Conclusions

1. In the context of research work carried out on the development of a new **HFRP** composite material recommended for the shipbuilding industry, meeting the relevant strength, operational, and economic requirements, and, in particular, environmental requirements, its use in the shipbuilding industry is envisaged in the sector of manufacturing selected recreational vessels (yachts and motorboats) and other floating products (buoys/floats, pontoons). Dedicated to each selected composite structure, the reinforcement of this material, in accordance with its individually applied technology, should be made of fabrics and/or emulsion mats of industrial hemp with variable weight and multidirectional arrangements (0, 90, ±45°) of the fibres of this plant.
2. Based on the flammability test of the **HFRP** composite reinforced with HFs, the possibility of quasi-complete utilisation of this material by means of energy recycling was verified and a fully positive result was obtained. When 100% of the composite mass was incinerated, 98% of thermal energy and 2% of ash were obtained, which is used as an important component of effective natural fertilisers (EU preferences regarding the circular economy (CEco)). It should be emphasised that in using the test method according to ISO 5660-1 [34], **the combustion heat of HFRP (183 MJ/m^2^) is 3.75 times higher than that of GFRP (48.8 MJ/m^2^)**. For comparison, the combustion heat of **HFRP** (31 MJ/kg) in relation to GFRP (21 MJ/kg), lignite (5.9–23 MJ/kg), and wood (18 MJ/kg) is approx. 1.5 times higher, and compared to hard coal (16.7–29.3 MJ/kg) and charcoal (30 MJ/kg), it is comparable **in favour of HFRP**.
3. A detailed analysis of the results of tests on the physical, mechanical, and morphological properties of new composite materials made it possible to determine the **SCC W_K_** for **HFRP** in relation to GFRP as **W_K_ = 1.66 (6)**, provided that the weight of the skin/shell of the selected floating object/structure is comparable.
4. In light of the stringent regulations enforced concerning the need to combat degradation and protect the environment (restrictive EU legal regulations in force since 2018), and the significant reduction in the amount of non-biodegradable plastic waste (in particular, GF in the shipbuilding industry in the sector of manufacturing selected recreational vessels (yachts and motorboats)), as well as other floating products (buoys/floats, pontoons), it is considered appropriate and reasonable to replace the commonly used non-ecological GFRP composite reinforced with glass fibres with a new-generation **HFRP** composite material reinforced with industrial hemp fibres (*Cannabis sativa* L.).

## Figures and Tables

**Table 1:** Designation of composite sample series taking into account the number of GF and HF reinforcement layers.

Number of Tested Reinforcement Layers K1–K6 and Additionally Determined Empirically KE1, KE2 Included in the Analysis Concerning the Determination of the Structural Correlation Coefficient W_K_	Designation of a Series of Samples
GF—*E*-glass—6 layers	**S1**
HF—industrial hemp—3 layers	**K1**
HF—industrial hemp—5 layers	**K2**
HF—industrial hemp—7 layers	**K3**
HF—industrial hemp—9 layers	**K4**
HF—industrial hemp—10 layers	**KE1**
HF—industrial hemp—11 layers	**K5**
HF—industrial hemp—12 layers	**KE2**
HF—industrial hemp—13 layers	**K6**

**Table 2:** Charpy impact strength values of **HFRP** and GFRP composites after one month of storage in dry containers in environment **(0)** (air), and then after three months of storage in representative aqueous environments **(1)–(4)**.

Designation of a Series of Samples/Aqueous Environments	Charpy Impact Strength, J/cm^2^				
	Air (0)	Demineralised Water (1)	Fresh Water (Lake Miedwie) (2)	Brackish Water—Salinity 7.8‰ (Baltic Sea) (3)	Salty Water—Salinity 38‰ (Adriatic Sea) (4)
**S1**	31.8 ± 0.9	22.7 ± 0.8	23.4 ± 0.6	23.8 ± 0.6	24.3 ± 0.6
**K1**	15.1 ± 1.2	16.1 ± 0.2	19.5 ± 1.0	15.2 ± 0.8	14.7 ± 0.6
**K2**	17.5 ± 1.1	22.0 ± 0.8	24.6 ± 0.7	18.6 ± 0.3	15.7 ± 0.9
**K3**	18.5 ± 0.4	23.7 ± 0.6	25.7 ± 0.4	22.6 ± 0.3	17.5 ± 0.4
**K4**	19.9 ± 1.1	30.9 ± 0.7	31.3 ± 0.6	31.6 ± 0.7	28.1 ± 0.2
**KE1**	20.5 ± 0.7	30.1 ± 0.7	32.4 ± 0.6	33.5 ± 0.6	29.3 ± 0.3
**K5**	21.1 ± 0.3	29.2 ± 0.6	33.4 ± 0.6	35.4 ± 0.5	30.5 ± 0.4
**KE2**	24.0 ± 1.0	29.1 ± 0.8	31.5 ± 0.9	35.1 ± 0.5	29.4 ± 0.5
**K6**	26.8 ± 1.7	29.0 ± 0.9	29.5 ± 1.2	34.7 ± 0.5	28.2 ± 0.5

**Table 3:** Analysis of the physical and mechanical properties of the tested **HFRP** and **GFRP** composites.

Test Type	Polymer Structural Composite	GFRP	HFRP
Physical	Density, g/cm^3^	1.6	1.2–1.3
	Water absorption, %	0.1	3.0–4.3
Environmental	Residue after burning, %	57.2 (GF—solid waste)	2.0 (HF—Ash)
Mechanical	Tensile elongation, %	2.4 ± 0.2	3.8 ± 0.2–4.4 ± 0.2
	Elongation under static bending, %	5.5 ± 0.5	2.2 ± 0.3–7.4 ± 0.2
	Maximum tensile forces, N	9541 ± 224	9209 ± 528
	Maximum static bending forces, N	525 ± 37	2228 ± 233
Impact tests were performed after storing the test samples for one month in dry trays with overall air access **(0)** and also after storing them for a further three months in representative aquatic environments **(1)**–**(4)**	Charpy impact strength in environment **(0)** (air), J/cm^2^	31.8 ± 0.9	15.1 ± 1.1–26.8 ± 1.7
	Charpy impact strength in environment **(1)** (demineralised water), J/cm^2^	22.7 ± 0.8	16.1 ± 0.2–30.9 ± 0.7
	Charpy impact strength in environment **(2)** (fresh water—Lake Miedwie), J/cm^2^	23.4 ± 0.6	19.5 ± 1.0–33.4 ± 0.6
	Charpy impact strength in environment **(3)** (brackish water—salinity 7.8‰—Baltic Sea), J/cm^2^	23.8 ± 0.6	15.2 ± 0.8–35.4 ± 0.5
	Charpy impact strength in environment **(4)** (saline water—salinity 38‰—Adriatic Sea), J/cm^2^	24.3 ± 0.6	14.7 ± 0.6–30.5 ± 0.4

## Data Availability

The original contributions presented in this study are included in the article. Further inquiries can be directed to the corresponding authors.
